# Influence of SARS-CoV-2 infection before and during organogenesis on embryo implantation and development outcomes: A prospective cohort observational study

**DOI:** 10.1371/journal.pone.0328743

**Published:** 2025-09-11

**Authors:** Yanping Li, Xuan Lu, Jing Fu, Fan Yang, Zenghui Mao, Hongqing Liao, Juan Zhang, Xianghong Huang, Qiong Zhang

**Affiliations:** 1 Center for Reproductive Medicine, Xiang-Ya hospital, Central South University, Changsha City, Hunan Province, China; 2 Center for Reproductive Medicine, Chenzhou No 1 People’s Hospital East Hospital, Chenzhou City, Hunan Province, China; 3 Center for Reproductive Medicine, Changsha Hospital for Maternal and Child Health Care of Hunan Normal University, Changsha City, Hunan Province, China; 4 Xinghui Reproductive Health hospital, Hengyang City, Hunan Province, China; 5 Center for Reproductive Medicine, Zhuzhou Central Hospital, Zhuzhou City, Hunan Province, China; 6 Center for Reproductive Medicine, Xiangtan Central hospital, Xiangtan City, Hunan Province, China; King Saud University / Zagazig University, EGYPT

## Abstract

**Background:**

Extensive research has demonstrated the detrimental effects of COVID-19 on maternal-fetal outcomes. However, few studies have examined the impact of SARS-CoV-2 infection before and during organogenesis on human embryo implantation and subsequent development. Additionally, the influence of SARS-CoV-2 on the endometrial microenvironment, which is critical for embryo implantation, remains poorly understood. This study seeks to address these gaps in knowledge.

**Methods and findings:**

We prospectively enrolled 971 participants undergoing frozen-thawed embryo transfer (FET) during the final two months of 2022, coinciding with the nationwide COVID19 outbreak following the end of China’s Zero-Covid policy. Patients undergoing FET during this period were at high risk of SARS-CoV-2 infection before and during organogenesis. Based on self-reported symptoms and nucleic acid testing, 520 individuals were confirmed to have SARS-CoV-2 infection, while 451 were uninfected. Consistent with existing literature, our study reinforced that SARS-CoV-2 infection negatively impacted pregnancy outcomes, as evidenced by reduced clinical pregnancy (52.69% vs. 76.50%, RR = 60.506, [95%CI, 0.259 ~ 0.452]) and live birth rates (46.54% vs. 60.09%, RR = 17.865, [95%CI, 0.448 ~ 0.746]), alongside an increase in obstetric complications (35.89% vs. 27.37%, RR = 4.380, [95%CI, 1.055 ~ 2.223]). Seven fetal congenital heart defects (CHDs) were observed in the infected group versus one in uninfected population. Bioinformatic analysis of endometrial mRNA profiles showed SARS-CoV-2 infection significantly downregulated key endometrial receptivity molecules, increased natural killer cell and mast cell infiltration, and disrupted the balance of cytokine and chemokine. Moreover, our findings demonstrated that SARS-CoV-2 infection downregulated the transcriptional activity of endometrial SLC6A, a serotonin transporter, and ErbB-2, a mediator of serotonin-regulated differentiation in cardiac development. This disruption in serotonin signaling may underlie the pathogenesis of congenital heart disease.

**Conclusions:**

SARS-CoV-2 infection before and during organogenesis negatively impacts embryo implantation and development, primarily through mechanisms involving compromised endometrial receptivity and disruption of the local immune microenvironment.

## Introduction

The Coronavirus Disease 2019 (COVID19) in pregnancy is associated with an increased risk of maternal/fetal morbidity and mortality [1,2], and fetal congenital malformations [3,4]. However, most evidence are based upon case-control studies, retrospective cohort studies and brief reports. Prospective cohort study is rare. Another overlooked factor is the timing factor, which is crucial in determining the occurrence and severity of teratogenic effects. Limited research has investigated the impact of SARS-CoV-2 infection during the critical stages of embryonic development on human embryo implantation and subsequent progression [5,6]. The embryo’s morphogenesis, from the blastocyst stage to organogenesis, makes this period particularly sensitive to environment influences [7]. Organogenesis takes place from weeks 3–8, during which the rudimentary structures of all major organs are formed. By week 8, organ systems have undergone differentiation and are primed for maturation. Once an organ or body part is formed, it is theoretically no longer susceptible to major birth defects.

Investigators have conducted exploratory research into the molecular mechanisms by which SARS-CoV-2 infection affects endometrial function. They view COVID-19 as an inflammatory process and identify key candidate genes and pathways to better understand how this zoonotic origin virus adversely influences human reproduction [8,9]. But they ignored the mRNA profiles of human endometrium undergo dynamic changes throughout the menstrual cycle in response to ovarian hormones [10]. In the following study, we adjusted the biopsy at a unified time point to align with the window of implantation (WOI), which can avoid errors caused by the cyclical changes of the endometrium. Furthermore, the underlying molecular mechanism for the teratogenicity of SARS-CoV-2 has not been explored [3,4]. Our research has made a meaningful attempt in this regard.

On December 7, 2022, the Zero-Covid policies were lifted in China [11]. Consequently, a nationwide SARS-CoV-2 outbreak lasted for a month [12]. Individuals who underwent embryo transfer (ET) in the two months before and after this period were at risk of SARS-CoV-2 infection before or during organogenesis. This presented a valuable opportunity to investigate the impact SARS-CoV-2 infection during this embryonic stage on subsequent embryo outcomes. To explore this, we conducted a prospective cohort study involving six fertility clinics across five cities in Hunan Province, China.

## Materials and methods

Only frozen-thawed embryo transfer (FET) cycles were enrolled in this prospective study. Given our previous study’s findings on the deleterious effect of SARS-CoV-2 on fresh embryo viability [13], we minimized potential confounding factors by including only thawed embryos that were cryopreserved during the Zero-COVID era. A total of 971 participants were recruited from 6 fertility clinics. Date of embryo transfer ranged from November 9 to December 24, 2022. Based on self-reported symptoms and nucleic acid testing for SARS-CoV-2, participants were categorized into two groups: infected and uninfected. During this Omicron-predominant COVID-19 pandemic, all 520 individuals experienced mild symptoms, recovered at home, and required no special treatment. With electronic consent, pregnant individuals were monitored monthly via phone, Wechat, or text message until pregnancy completion.

To investigate the influence of SARS-CoV-2 infection on endometrial receptivity and the embryo development milieu, we analyzed endometrial mRNA profile datasets during WOI from an ongoing registered clinical trial (ChiCTR2100049841), rather than conducting RNA sequencing ourselves.

### Ethical statement

On December 12^th^ 2022, an approval was obtained from the Ethic Committee of Reproductive Medicine, Xiang-Ya Hospital, Central South University, China. On February 6^th^, 2023, the clinical trial was retrospectively registered at the Chinese Clinical Trial Registry (ChiCTR2300068080). Electronic signatures were obtained from all participants prior to data collection and analysis.

The first patient was recruited on December 13, 2022, after obtaining the ethic approval. She had undergone FET on November 14, 2022, and was diagnosed with SARS-CoV-2 infection on December 10, 2022 (Day 26 post-ET). Given the urgency of the study and the narrow time window for positive COVID19 cases, patient recruitment began immediately after ethical approval, with some patients enrolled before obtaining the online ethics registration number on February 6, 2023.

To minimize the risk of SARS-CoV-2 infection during early pregnancy, endometrial preparation for FET was suspended from December 7, 2022. Patients already undergoing endometrial preparation were advised to discontinue treatment if infection. Uninfected patients could proceed with ET after being informed of the potential risks associated with post-ET SARS-CoV-2 infection.

### Sample size calculation

The previous average CPR of assisted reproductive technology in China was 50%. A priori sample size calculation estimated that minimum of 800 participants (400 each group) would provide 80% power to test the negative influence of SARS-CoV-2 infection on CPR assuming a 10% difference and an alpha value of 0.05. Anticipating a 10% dropout rate, at least 880 participants were required for recruitment.

### Inclusion and exclusion criteria

Inclusion criteria: FET cycle, aged 20–40 years, BMI ranged 18–30, endometrial thickness ≥7.0 mm at progesterone start.

Exclusion criteria: recurrent implantation failure, recurrent pregnancy loss, pre-implantation genetic testing, congenital uterine anomaly, intrauterine adhesions, smoking, preexisting hypertensive disorder, diabetes mellitus and autoimmune disease, recurrent SARS-CoV-2 infection during pregnancy.

### Exposure

Based on self-reported symptoms and nucleic acid testing for SARS-CoV-2 on nasopharyngeal swabs, participants were divided into two groups: infected and uninfected.

### Endometrium preparation

The endometrium was uniformly prepared using hormone replacement therapy. Exogenous estrogen was supplemented for 2 weeks. When endometrial thickness reached 7.0 mm or greater, progesterone was added to transform the proliferative endometrium into the secretory structure.

### Endometrium sampling

Written informed consent was obtained before endometrial biopsy. No contraindication to operation was confirmed. Patient lied in a lithotomy position. Cervix was cleaned with iodine. A Pipelle sampler (Aimu, Liaoning, China) was inserted through the cervix into the uterine cavity, avoiding contact with the fundus. Created negative pressure via pulling back the inner core. Rotated the Pipelle gently and moved back and forth to aspirate adequate tissue sample. Withdrew the Pipelle from the uterine cavity. Endometrial specimens were rinsed in 0.9% NaCl to remove mucus, then immersed in fixative solution for preservation and subsequent sequencing.

### mRNA sequencing

The mRNA sequencing was performed by Yikon Genomics Company, Ltd. RNA extraction, quality assessment, mRNA enrichment, library preparation and sequencing were introduced in our previous study [14].

### Main outcome measures

The primary outcomes were pregnancy outcomes, including clinical pregnancy rate (CPR), embryo implantation rate (IR), ongoing pregnancy rate (OPR), live birth rate (LBR) and incidence of obstetric complications.

The secondary outcome was the alterations of endometrial mRNA profiles in the context of SARS-CoV-2 infection.

Clinical pregnancy was defined as the presence of an intrauterine gestational sac with a heartbeat at 4–5 weeks post-ET. Ongoing pregnancy refers to the viable pregnancy ≥ 20 gestational weeks. Live birth means the birth of living infant showed sign of life, ≥ 28 gestational weeks regardless the birthweight.

### Statistical analysis

Statistical analysis was conducted using IBM SPSS statistic, version 25.0. Chi-square (χ^2^) test was used to compare dichotomous outcomes. Normally distributed data were expressed as mean ± standard deviation. Differences were tested by two-tailed student’s t-test. The values p < 0.05 were considered statistically significant. Bivariate logistic regression analysis was employed to determine independent variables influencing pregnancy outcomes.

CIBERSORTx was employed for inference of cell-type specific gene expression profiles in endometrial tissue [15].

## Results

**Fig 1:**
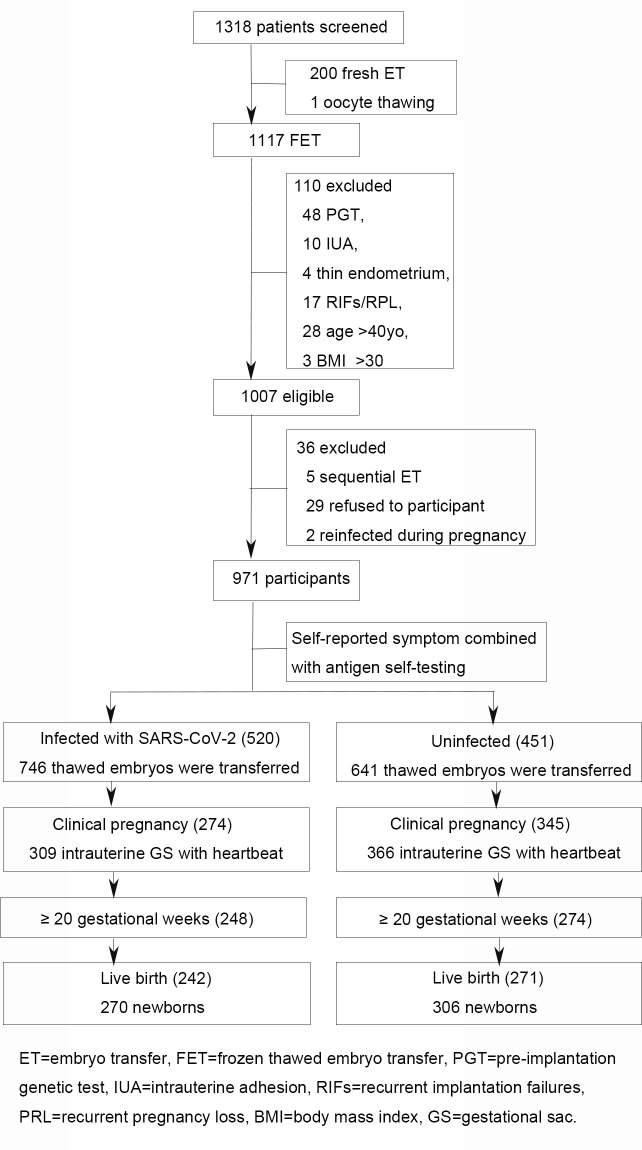
Flowchart of participant recruitment, assessment and follow-up. During the last two months of 2022, 1318 patients underwent embryo transfer. After a series of filtration process steps, 971 participants met inclusion/exclusion criteria. Based on SARS-CoV-2 nucleic acid testing, participants were divided into two groups: 520 infected and 451 uninfected. In the infected group, 274 participants achieved clinical pregnancy, resulting in 242 live births. In uninfected group, 345 conceived successfully and 271 gave birth.

At the beginning, 1318 participants were screened: 744 in Xiang-Ya Hospital, Changsha City, 203 in Chenzhou No 1 People’s Hospital East Hospital, Chenzhou City, 136 in Changsha Hospital for Maternal and Child Health Care of Hunan Normal University, Changsha City, 127 in Xinghui Reproductive Health hospital, Hengyang City, 76 in Zhuzhou Central hospital, Zhuzhou City, 32 in Xiangtan Central hospital, Xiangtan City. After a series of filtration process steps, 971 participants met all inclusion and exclusion criteria. Of them, 520 were diagnosed with SARS-CoV-2 infection, while 451 were uninfected. All the infectious cases were mild illness without hospitalization. Every participant gave us a full cooperation in follow-up process. Flowchart of participant recruitment, assessment and follow-up was presented in Fig 1.

### The baseline characteristics of participants

As shown in Table 1, similar demographic characteristics were observed between the two groups. The mean endometrial thickness on the first day of progesterone administration was 10.3 mm in both groups. Two-thirds participants in both groups underwent single embryo transfer (SET). Blastocysts vitrified on the 5^th^ or 6^th^ day post-oocyte retrieval were transferred for 70.0% in infected and 71.8% in uninfected group respectively.

**Table 1:** Demographic and cycle characteristics for all participants.

	Infected	Uninfected	*p*-value	95%CI/ RR
No.	520	451		
Demographic characteristics				
Age	31.92 ± 7.85	32.51 ± 4.97	0.158	−0.214 ~ 0.038
BMI	21.78 ± 2.73	22.25 ± 3.16	0.075	−0.322 ~ 0.015
Infertile period	4.36 ± 4.00	4.76 ± 4.05	0.130	−0.224 ~ 0.029
Primary infertility	193	219	0.456	0.593
Secondary infertility	227	232		
Endometrial preparation				
HRT cycle	384	350	0.178	1.856
Non-HRT cycle	136	101		
Peak thickness	10.25 ± 1.69	10.28 ± 1.60	0.795	−0.245 ~ 0.109
Embryo evaluation				
Number	1.44 ± 0.50	1.37 ± 0.48	0.037	0.004 ~ 0.128
SET cycle	292	283	0.042	4.360
DET cycle	228	168		
Development stage				
Cleavage	156	127	0.571	0.394
Blastocyst	364	324		

BMI = body mass index, HRT = hormone replacement therapy, SET = single embryo transfer, DET = double embryo transfer, 95%CI = 95% confidence interval, RR = rate ratio.

### Effect of maternal SARS-CoV-2 infection on pregnancy outcomes

Adverse pregnancy outcomes were observed in individuals infected with SARS-CoV-2. The CPR was 52.69% (274/520) compared to 76.50% (345/451) (RR = 60.506, [95%CI, 0.259 ~ 0.452]). The IR was 41.42% (355/748) versus 59.13% (366/619) (RR = 44.817, [95%CI, 0.284 ~ 0.490]). The OPR was 47.69% versus 60.75% (RR = 16.641, [95%CI, 0.456 ~ 0.761]). And the LBR was 46.54% versus 60.09% (RR = 17.865, [95%CI, 0.448 ~ 0.746]).

**Fig 2:**
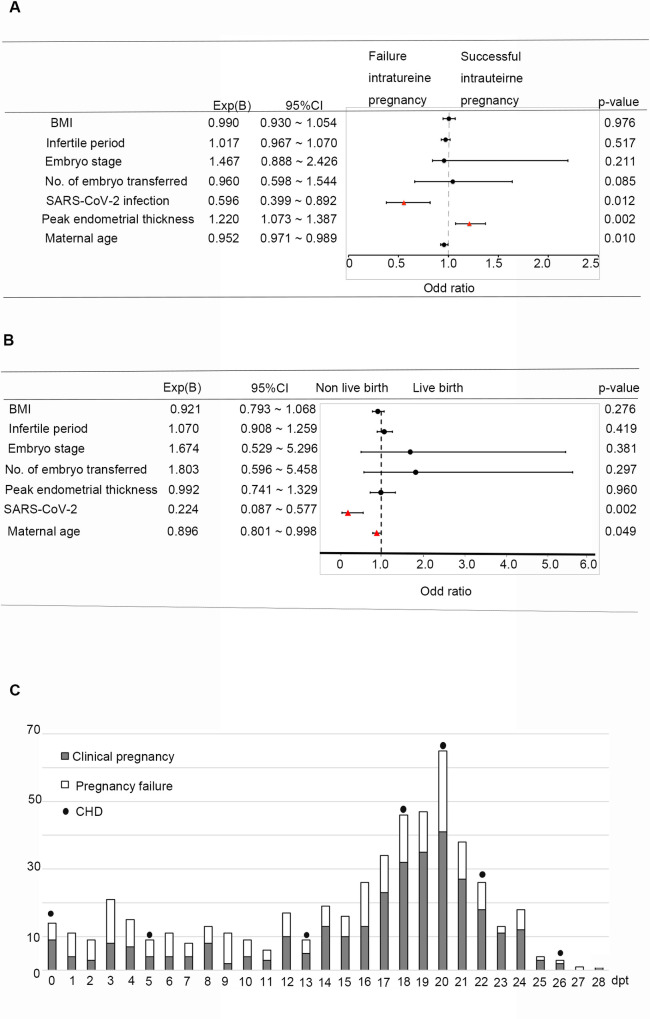
Effect of maternal SARS-CoV-2 infection on pregnancy outcomes. **A.** Bivariate logistic regression analysis identified maternal age, endometrial thickness and SARS-CoV-2 infection were the three significant predictors for a clinical pregnancy. **B.** For live birth prediction, maternal age and SARS-CoV-2 infection were the two independent factors. **C.** Seven congenital heart defects (CHDs) were diagnosed in cases with maternal COVID-19. These CHDs were evenly distributed in relation to the timing of maternal COVID-19 diagnosis. (dpt = days post embryo transfer).

We assessed the incidence of obstetric complications occurred after 20 weeks of pregnancy and found 35.89% (89/248) infected individuals experienced maternal/perinatal complications, significantly higher than 27.37% (75/274) observed in uninfected individuals (RR = 4.380, [95%CI, 1.055 ~ 2.223]). The teratogenic effect of SARS-CoV-2 infection before and during organogenesis is another important focus of our research. Congenital heart defects (CHDs) were the most commonly observed anomaly, occurring in 8 cases (Table 2). These 7 CHDs in infected group were evenly distributed relative to the timing of maternal COVID-19 diagnosis (Fig 2C). Additional congenital malformations included cleft lip and palate (2 fetuses), and anal atresia (1 fetus).

**Table 2:** Pregnancy outcomes in infected and uninfected participants.

	Infected	Uninfected	*p*-value	RR	95%CI
No. of participants	520	451			
No. of embryo transferred	748	619			
Clinical pregnancy	274	345	<0.001	60.506	0.259 ~ 0.452
CPR	52.69%	76.50%			
GS with heartbeat	309	366	<0.001	44.817	0.284 ~ 0.490
IR	41.13%	59.13%			
Ongoing pregnancy	248	274	<0.001	16.641	0.456 ~ 0.761
OPR	47.69%	60.75%			
Live birth	242	271	<0.001	17.865	0.448 ~ 0.746
LBR	46.54%	60.09%			
Take-home baby	270	306	<0.001	25.700	0.453 ~ 1.326
THBR	36.19%	49.84%			
Complications	89	75	0.038	4.380	1.055 ~ 2.223
Rate	35.89%	27.37%			
Maternal morbidities					
HDP	20	9			
GDM	20	11			
Placenta previa	6	2			
PROM	3	5			
others	9	2			
Perinatal complications					
Preterm birth	37	47			
LBW	21	38			
IUFD	5	4			
CHD	7	1			
CLP	2	0			
others	2	0			

CPR = clinical pregnancy rate, IR = embryo implantation rate, OPR = ongoing pregnancy rate, HDP = hypertensive disorders of pregnancy, CHD = congenital heart disease, LBR = live birth rate, THBR = take-home baby rate, HDP = hypertensive disorders of pregnancy, GDM = gestational diabetes mellitus, PROM = premature rupture of membrane, LBW = low birth weight, CHD = congenital heart disease, IUFD = intrauterine fetal demise, CLP = cleft lip and/or palate, RR = rate ratio, 95%CI = 95% confidence interval. Obstetric complications: If a patient experienced multiple complications, including those related to the fetus, count it as one obstetric complication.

Bivariate logistic regression analysis was employed to identified the most significant factors associated with pregnant outcomes. We found younger maternal age, thicker endometrium, and the absence of SARS-CoV-2 infection were protective factors for achieving a successful clinical pregnancy. The predictive factors were listed in order of their strength, as following: endometrium thickness (Exp(B)=1.220, [95%CI, 1.073 to 1.387]), maternal age (Exp(B)=0.952, [95%CI, 0.917 to 0.989]) and absence of SARS-CoV-2 infection (Exp(B)=0.596, [95%CI, 0.399 to 0.892]) (Fig 2A). For live birth, younger maternal age and absence of SARS-CoV-2 infection were protective factors. And the predictive powers were: maternal age (Exp(B)=0.896, [95%CI, 0.801 to 0.998]), absence of SARS-CoV-2 infection Exp(B)=0.224, [95%CI, 0.087 to 0.577] (Fig 2B).

### Endometrium played a dual role in the context of SARS-CoV-2 infection

Under physiological conditions, the maternal-fetal interface constitutes a physical and immune defense shielding the embryo from pathogens in maternal circulation. In the context of SARS-CoV-2 infection, endometrial immunity played a dual role, acting as both a protective ally and a potential adversary.

**Fig 3:**
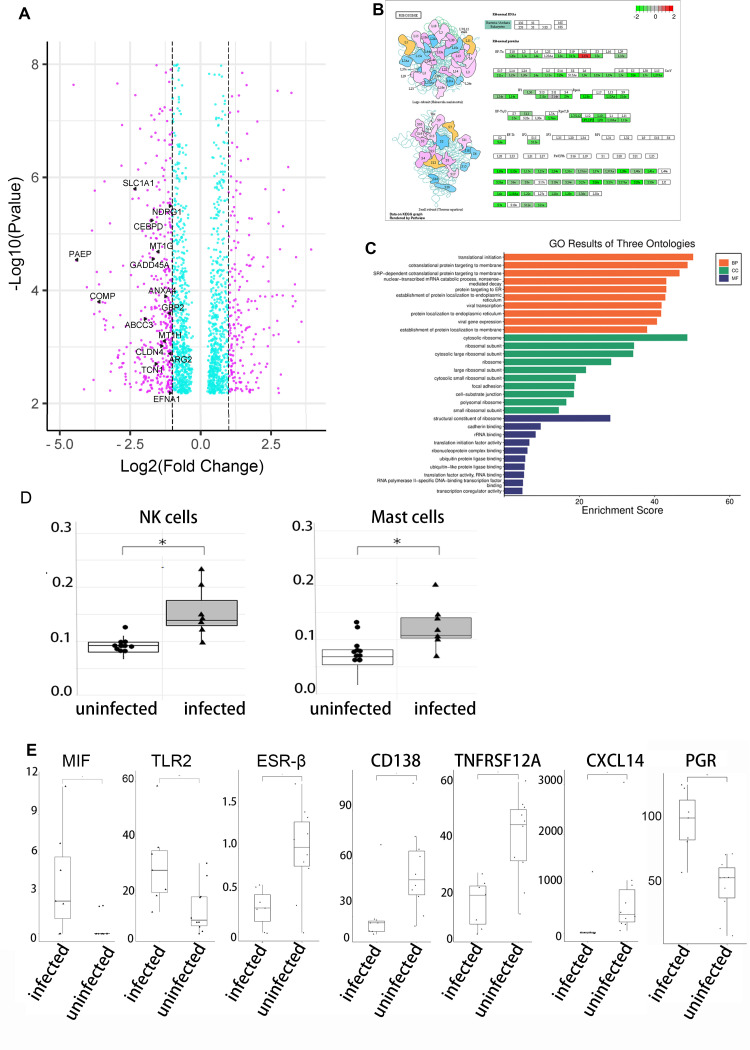
Impact of SARS-CoV-2 infection on endometrial mRNA profiles. A. A total of 2217 DEGs (differentially expressed genes) were identified in COVID-19 individuals. 963 up-regulated and 1254 down-regulated. Biomarkers of receptive endometrium were generally down regulated, such as *PAEP, CLDN4, ABCC3, ANXA4*, and so on. B. Numerous ribosome units were downregulated after SARS-CoV-2 infection. C. GO and KEGG analysis detected translational initiation, protein sorting and focal adhesion were disrupted, while viral transcription and viral gene expression were activated. D. CIBERSORTx exhibited an improved endometrial infiltration of NK (natural killer) cell and mast cell in the context of SARS-CoV-2 infection. E. SARS-CoV-2 changed the expression of MIF, TLR2, ESR2, CD138, TNFRSF12A, CXCL14 and PGR.

We analyzed 17 endometrial mRNA profile datasets, including 10 from uninfected and 7 from infected individuals. Bioinformatic analysis deciphered a robust alteration in endometrial mRNA profiles following SARS-CoV-2 infection. Through calculating differential gene expression as a log2 fold change (log_2_FC) >1 and a false discovery rate (FDR) <0.01, we identified 2217 differentially expressed genes (DEGs), 963 up-regulated and 1254 down-regulated in COVID-19 individuals (Fig 3A). Through the Gene Expression Omnibus (GO) and Kyoto Encyclopedia of Genes and Genomes (KEGG) pathway enrichment analysis, numerous ribosome protein genes were detected to be downregulated by SARS-CoV-2 infection (Fig 3B). The biological processes of translational initiation, protein sorting and focal adhesion were significantly disrupted, while viral transcription and viral gene expression were activated (Fig 3C). Biomarkers of receptive endometrium were generally down regulated in individuals with COVID-19 (Fig 3A).

Employing CIBERSORTx to capture cell-type-specific gene expression from mRNA profiles, 22 immunocyte subsets were identified in endometrial specimens. Silico cell enumeration and purification demonstrated an improved infiltration of natural killer (NK) cell and mast cell following SARS-CoV-2 infection (Fig 3D).

A total of 88 cytokine and chemokine genes were analyzed in the endometrium. Up-regulated MIF (macrophage migration inhibitory factor) and TLR2 (toll-like receptor 2), and down-regulated CD138 (syndecan-1), TNFRSF12A (TNF receptor superfamily member 12A) and CXCL14 (C-X-C motif ligand 14) were determined in endometrial specimens collected from COVID 19 individuals (Fig 3E).

## Discussion

SARS-CoV was firstly identified in 2002 [16]. This zoonotic origin virus (transmission from animals to humans) spreads via airborne particle and evolves as changes in genetic code occur during replication of the genome. Vice versa, it has the potential of reverse zoonosis (transmission from humans to animals) and maintains infectious agents in nature. Till now, neither the natural reservoir host nor the intermediate host for SARS-CoV is identified [17]. Risks for new outbreak of SARS-CoV remain high. Before vaccine effectiveness defeats virus mutation [18], the future outbreak of this zoonotic disease is inevitable and unpredictable. A comprehensive understanding of the impact of SARS-CoV-2 infection on human reproduction is essential, including the mechanisms through which it affects mothers and offspring. Additionally, identifying potential biomarkers for monitoring or prediction and developing effective therapeutic interventions is crucial.

Consistent with published studies [3,19], this prospective observational cohort study supports maternal SARS-CoV-2 infection hampers successful rate of embryo implantation and increases incidence of obstetric complications and risk of congenital malformations. The total clinical pregnancy rate in the study was 63.75% (619/971), significantly exceeding the national average. Two rational explanations may account for this observation. First, the recruited individuals were likely to have a favorable prognosis, which could contribute to better outcomes. Second, patients with suboptimal embryo quality were more likely to have their embryo transfer postponed until after the resolution of COVID-19.

In the study, we make attempt to explore the molecular mechanisms underpinning the adverse pregnant outcomes in COVID-19. Our results show SARS-CoV-2 infection impairs endometrial receptivity through the following three ways. SARS-CoV-2 downs regulate the gene expression of key biomarkers for endometrial receptivity, such as PAEP, ANXA4, GADD45A, ARG2 and so on [20]. SARS-CoV-2 induces inappropriate immune response for hindering embryo implantation [21,22]. SARS-CoV-2 enhances endometrial infiltration of NK cells and mast cells. Uterine mast cells are located in close proximity to the smooth muscle of the myometrium [23]. SARS-CoV-2 infection triggers mast cells degranulation [24], releasing cytokines and/or chemokines [25,26] and driving NK cells recruitment [27]. Additionally, we found SARS-CoV-2 changed the expression of MIF, TLR2, ESR-β, CD138, TNFRSF12A, CXCL14 and PGR. MIF inhibits apoptosis of immune cells sustaining strong inflammatory responses [28]. TLR2 senses to the SARS-CoV-2 envelope protein inducing transcription of genes encoding pro-inflammatory cytokines [29]. Endometrial estrogen receptor-beta (ESR-β) [30] and progesterone receptor (PGR) [31] serve as critical signaling molecules essential for endometrial receptivity.

Consistent with published research [3], our clinical data indicate an elevated risk of CHDs in offspring following SARS-CoV-2 infection before and during organogenesis. It is well-known that early embryos use serotonin (5-hydrooxytryptamine) (5-HT) to regulate cardiac development [32]. There are three main areas of 5-HT in body: the intestinal wall, platelets and central nervous system [33]. Prior to the proliferation of fetal neural progenitor cells (at 4 weeks of embryonic age) [34], platelet-derived maternal 5-HT is the critical concentration required for cardiac myocyte differentiation and proliferation [35]. A published study has highlighted a reduction in serotonin (5-HT) as a consequence of SARS-CoV-2 infection [36]. Our RNA-seq data demonstrates endometrial expression of SLC6A (5-HT transporter [37])(log_2_FC = −1.659 p = 0.00036) and ErbB-2 (mediate 5-HT control differentiation in developing heart [35]) (log_2_FC = −0.613 p = 0.006) was significantly downregulated following SARS-CoV-2 infection. This downregulation is implicated in the disruption of serotonin (5-HT) signaling pathways, which may underlie the pathogenesis of congenital heart disease.

How dose SARS-CoV-2 impact the endometrium? To date, there is no evidence supporting the in vivo infection of the human endometrium by SARS-CoV-2 [9,38]. Our RNA-seq data suggest that SARS-CoV-2 infection leads to significant downregulation of ribosome subunits biogenesis, as well as considerable disruptions in translational initiation, protein sorting, and focal adhesion. A review of existing literature indicates that non-structural protein 1 (NSP1) may play a role in mediating the effect of SARS-CoV-2 on endometrial function [39]. But how is NSP1 delivered into endometrial cells? We hypothesize NSP1 may enter endometrial cells through specialized cargo mechanisms. COVID-19-derived plasma exosomes, for example, can protect SARS-CoV-2 viral particles from RNase degradation and facilitate the transfer of double-stranded RNA into recipient cells, thereby initiating signaling pathways [40,41]. These exosomes could also aid in the transport of NSP1 into endometrial cells.

Several limitations of the study need to be mentioned. A key limitation of this study is the absence of gestational or placental tissue samples from pregnancy losses for RNA sequencing, which restricts our ability to directly assess molecular changes associated with adverse outcomes. Further research would benefit from longitudinal studies that include comprehensive tissue sampling to investigate the effects of SARS-CoV-2 on placental development and function, and to guide targeted clinical interventions. Additionally, the sample size was insufficient to fully assess SARS-Cov-2’s teratogenic effects, and its impact on the nervous system remains unevaluated. Furthermore, potential confounding factors such as maternal comorbidities, medication use, and socioeconomic variables were not fully accounted for, which may have influenced the observation outcomes.

This study demonstrates SARS-CoV-2 infection before and during organogenesis adversely affects embryo implantation, elevates the risk of pregnancy complications, and increases the likelihood of congenital malformations in offspring. When providing patient counseling, careful consideration of the timing of SARS-CoV-2 infection is essential to understanding its impact pregnancy outcomes.

## Conclusions

SARS-CoV-2 infection before and during organogenesis negatively impacts embryo implantation and development, primarily through mechanisms involving compromised endometrial receptivity and disruption of the local immune microenvironment.

**Capsule:**

SARS-CoV-2 infection before and during organogenesis disrupts embryo implantation and development by impairing endometrial receptivity and altering the local immune microenvironment.

## Supporting information

S1 File STROBE checklist cohort. (DOCX)
